# Addressing the Compartmentalization of Specific Integrin Heterodimers in Mouse Sperm

**DOI:** 10.3390/ijms20051004

**Published:** 2019-02-26

**Authors:** Michaela Frolikova, Eliska Valaskova, Jiri Cerny, Audrey Lumeau, Natasa Sebkova, Veronika Palenikova, Noemi Sanchez-Hernandez, Alzbeta Pohlova, Pavla Manaskova-Postlerova, Katerina Dvorakova-Hortova

**Affiliations:** 1 Laboratory of Reproductive Biology, Institute of Biotechnology of the Czech Academy of Sciences, v.v.i., BIOCEV, Prumyslova 595, 25250 Vestec, Czech Republic; michaela.frolikova@ibt.cas.cz (M.F.); eliska.valaskova@ibt.cas.cz (E.V.); audrey.lumeau@etu.univ-orleans.fr (A.L.); natasa.sebkova@ibt.cas.cz (N.S.); veronika.palenikova@ibt.cas.cz (V.P.); Noemi.Sanchez@ibt.cas.cz (N.S.-H.); Alzbeta.Pohlova@ibt.cas.cz (A.P.); pavla.postlerova@ibt.cas.cz (P.M.-P.); 2 Laboratory of Structural Bioinformatics of Proteins, Institute of Biotechnology of the Czech Academy of Sciences, v.v.i., BIOCEV, Prumyslova 595, 25250 Vestec, Czech Republic; jiri.cerny@ibt.cas.cz; 3 Group of Cell Biology and Innovative Therapies, Centre for Molecular Biophysics, University of Orleans, UPR4301, 45071 Orleans, France; 4 Department of Biochemistry, Faculty of Science, Charles University, Vinicna 7, 12844 Prague 2, Czech Republic; 5 Department of Veterinary Sciences, Faculty of Agrobiology, Food and Natural Resources, University of Life Sciences Prague, Kamycka 129, 16500 Prague 6, Czech Republic; 6 Department of Zoology, Faculty of Science, Charles University, Vinicna 7, 12844 Prague 2, Czech Republic

**Keywords:** sperm head, integrins, integrin heterodimers, α6β4, α3β1, α6β1

## Abstract

Integrins are transmembrane cell receptors involved in two crucial mechanisms for successful fertilization, namely, mammalian intracellular signaling and cell adhesion. Integrins α6β4, α3β1 and α6β1 are three major laminin receptors expressed on the surface of mammalian cells including gametes, and the presence of individual integrin subunits α3, α6, β1 and β4 has been previously detected in mammalian sperm. However, to date, proof of the existence of individual heterodimer pairs in sperm and their detailed localization is missing. The major conclusion of this study is evidence that the β4 integrin subunit is expressed in mouse sperm and that it pairs with subunit α6; additionally, there is a detailed identification of integrin heterodimer pairs across individual membranes in an intact mouse sperm head. We also demonstrate the existence of β4 integrin mRNAs in round spermatids and spermatogonia by q-RT-PCR, which was further supported by sequencing the PCR products. Using super-resolution microscopy accompanied by colocalization analysis, we located integrin subunits as follows: α6/β4-inner apical acrosomal membrane and equatorial segment; α3, α6/β1, β4-plasma membrane overlaying the apical acrosome; and α3/β1-outer acrosomal membrane. The existence of α6β4, α3β1 and α6β1 heterodimers was further confirmed by proximity ligation assay (PLA). In conclusion, we delivered detailed characterization of α3, α6, β1 and β4 integrin subunits, showing their presence in distinct compartments of the intact mouse sperm head. Moreover, we identified sperm-specific localization for heterodimers α6β4, α3β1 and α6β1, and their membrane compartmentalization and the presented data show a complexity of membranes overlaying specialized microdomain structures in the sperm head. Their different protein compositions of these individual membrane rafts may play a specialized role, based on their involvement in sperm-epithelium and sperm-egg interaction.

## 1. Introduction

When sperm meets egg during fertilization, cell-cell interaction is a very complex molecular event mediated by a series of mutual protein contacts. Sperm-egg recognition, adhesion and fusion are an important part of this process, and sperm membranes, with their associated proteins, play a crucial role. Proteins localized on an egg surface form an extensive network called the tetraspanin web, where they associate and cooperate. Highly significant members of the egg tetraspanin web are integrins. The integrins are transmembrane proteins that, in somatic cells, participate in many cell-cell and cell-extracellular matrix interactions. These membrane receptors are an important part of the signaling pathway, and significantly, they have the ability to transmit signals in both directions—into and out of a cell. The integrins are also able to associate with other membrane receptors in multi-molecular complexes that participate in cell activation. In the egg, integrins are the binding partners of sperm surface proteins [1]; they associate with other tetraspanins, mediate connections between them [2,3] and interact with actin cytoskeleton [4,5]. To date, many molecules present on the egg were also discovered on sperm; therefore, the existence of a similar network to the egg tetraspanin web is predicted to exist on sperm. 

The integrins are transmembrane heterodimers; they are comprised of non-covalently associated α and β subunits. Nearly each known integrin subunit consists of an extracellular part that contains most protein polypeptides (more than 1600), one transmembrane domain and short cytoplasmic tail [6,7] usually of two domains range of 20–75 amino acids [6,8]. A substantial homology exists between cytoplasmic tails of individual β subunits in contrast to α subunits where this part differs between individual subunits; however, a part responsible for the interaction with the β subunit (containing GFFKR motif) is conserved in all cytoplasmic tails of α subunits [8,9,10,11]. This particular motif is essential for the heterodimerization of integrins [10], and it plays an important role in the maintenance of the integrin heterodimer in an inactive state [11]. On the other hand, in β subunits, there is a conserved Arginine/Lysine finger in the extracellular part, which is crucial for integrin subunit interaction resulting in heterodimerization. However, β4 represents an exception to the above. Not only is the cytoplasmic tail of β4 subunit large, consisting of approximately 1000 amino acids [6,8] but β4 also lacks the Arg/Lys finger that is replaced by Alanine and followed by Asparagine/Valine insertion unique for this particular integrin subunit [7]. The 3D structure of integrin α/β heterodimers in a resting state is very similar for both main groups of complexes with or without the α-I domain inserted into the propeller domain, as can be seen for example in existing crystal structures of αXβ2 (PDB ID 4neh; [12]) or αIIbβ3 (PDB ID 3fcs; [13]). The structure of an activated integrin complex in its open state can be represented by the αIIBβ3 crystal structure (PDB ID 3fcu; [13]). Interestingly, up to now, only the α6 subunit has been identified to form a functional heterodimer with β4 [7,14]. Moreover, all integrins mediate interaction with the actin cytoskeleton; however, α6β4 also interact mainly via plectin with an intermediate filament such as keratin [15,16], which does not compromise its ability to associate with actin filaments and microtubules [16,17].

To date, 18 α and 6 β subunits and their 24 different heterodimer combinations [6,8] have been identified in mammalian somatic cells. According to ligand specificity, these heterodimers are divided into four group-aminin receptors, collagen receptors, leukocyte-specific receptors and RGD receptors. In the case of sperm, there are α3, α4, α5, α6, αV, β1, β3 and β4 subunits known to be expressed [18,19,20,21,22,23,24]; however, not every subunit was described in all commonly-studied species, e.g. β4 was reported only in human sperm [19]. Additionally, knowledge about individual heterodimer pairs in sperm and their detailed localization is completely missing. Therefore, the role of integrins in reproduction remains unclear. 

The goal of this study was to characterize a presence of α3, α6, β1 and β4 integrin subunits in the intact mouse sperm head and identify a sperm-specific localization for heterodimers α6β4, α3β1 and α6β1. Moreover, the study presents an integrin specific membrane compartmentalization and addresses a complexity of membranes overlaying specialized microdomain structures in the sperm head. 

## 2. Results

**Figure 1:**
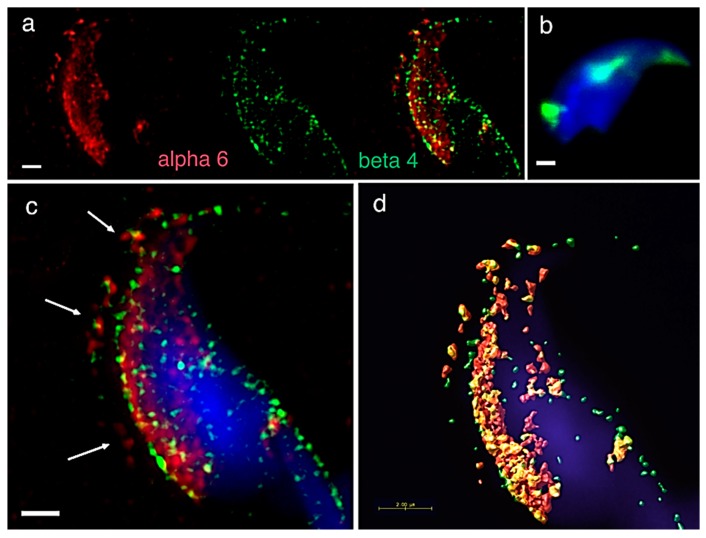
Mutual localization of the α6 and β4 integrin subunit and a presence of α6β4 heterodimer revealed by SIM and PLA. (**a**) α6 (red) and β4 (green) are localized in the plasma membrane overlying the acrosomal cap (for details see panel **c**, white arrows), apical hook and equatorial segment in an intact sperm head. (**b**) PLA confirmed the presence of the α6β4 heterodimer in depicted locations (**a**). (**c**) SIM dual-colour imaging showing merged image of α6 (red) and β4 (green) localized in the plasma membrane (white arrows). (**d**) Huygens software was used for a better visualization of the colocalization area (yellow). Colocalization maps are based on Pearson’s correlation coefficient. Nucleus is visualized with Dapi (blue). Scale bar represents 1 µm (**a**–**c**) and 2 µm (**d**).

A precise localization of α3, α6 and β1 integrin subunit was reported previously [24], showing that apart from their expression in plasma and/or acrosomal membranes, the α6 but not β1 subunit occupies the equatorial segment in a very distinctive pattern (Figure 1a). In order to find its binding heterodimer partner in mouse sperm, we addressed in great detail the possible presence of the β4 subunit. Using dual immunofluorescent staining visualized by 3D super resolution microscopy, we visualised the localization of β4 (Figure 1a) identical to the one of α6, covering the plasma membrane and also the equatorial segment. We also used dual immunofluorescent staining and confocal imaging to obtain data for colocalization analysis. Pearson’s correlation coefficient was used for the evaluation of the colocalization of the studied proteins. The resulting average value of Pearson’s correlation coefficient of α6 and β4 integrin subunits was 0.6558 ± 0.05, which means a high rate of colocalization. The mutual interaction was confirmed by PLA (Proximity Ligation Assay), which confirmed the existence of the α6β4 heterodimer. PLA is an immunoassay that detects proteins in proximity shorter than 40 nm, which is a distance when proteins interact between each other. PLA is commonly used to confirm or rule out predicted protein-protein interactions. Structural illumination microscopy (SIM) dual color imaging was used to create a colocalization map based on Pearson’s correlation coefficient for better visualization of the colocalization area of the identified integrin subunits (Figure 1c,d). 

To assess the expression of the β4 integrin subunit (*Itg β4*) in the mouse sperm, we prepared testicular elutriation fractions. To determine enrichment of individual fractions by relevant sperm cell type, we performed q-RT-PCR and defined each fraction by specific gene markers. We targeted the germinal cells using gene markers for spermatogonia, primary spermatocytes, round spermatids, round/elongating spermatids, Leydig and Sertoli cells summarized in the Supplementary Table S1. 

After characterization of the elutriation fraction, we analyzed the expression of *β4 integrin*. We showed mRNA expression in all five fractions, mainly at the spermatogonia and round spermatids (Table 1). 

**Figure 2:**
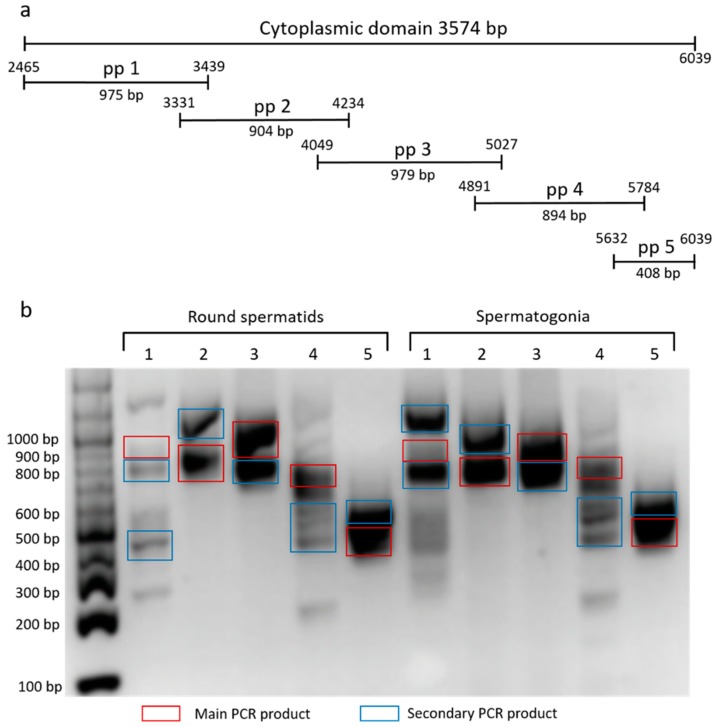
(**a**) Primers designing and (**b**) agarose gel electrophoresis of PCR products involving in the cytoplasmic domain of β4 integrin. 1–5 PCR products amplified by primer pairs (pp) in mRNA sperm samples after elutriation.

To characterize the cytoplasmic domain of β4 integrin in mouse germinal cells, we used set of primers pair (pp) corresponding to a specific region of the domain (Figure 2a). We amplified the fraction consisting of round spermatids and spermatogonia, and ran products on DNA agarose gel (Figure 2b). Main and marked secondary PCR products were sequenced, involving two described (NCBI Reference Sequences: NM_001005608.2 and NM_133663.2) and six predicted *β4 integrin* transcript variants X1-6 (NCBI Reference Sequences: XM_017314349.1, XM_017314350.1, XM_017314351.1, XM_006532572.3, XM_006532573.3 and XM_006532574.2). The secondary PCR products represent not only shorter *β4 integrin* transcripts, but also a longer one (Figure 2b).

**Figure 3:**
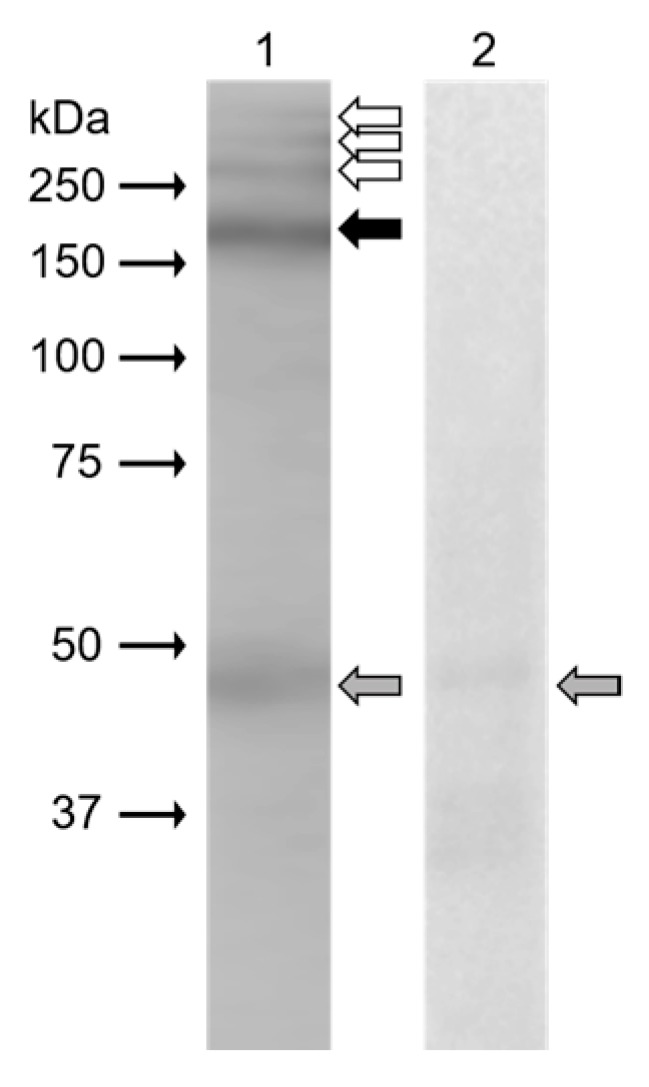
Western blot immunodetection of the β4 integrin in protein extract from mouse epididymal sperm with mouse monoclonal anti-β4 integrin (sc-13543) antibody; (1) antibody reaction in protein extract from mouse sperm, (2) negative control with mouse IgG; detection of 200 kDa protein band corresponds to β4 integrin (black arrow), possible high molecular weight isoforms (white arrows), non-specific reaction (grey arrows).

In Western blot analysis (Figure 3), monoclonal antibody anti-β4 integrin (sc-13543, against full-length integrin β4 of human origin) clearly recognized protein band (black arrow) of 200 kDa in the extract from mouse epididymal sperm. Weak reaction was displayed in protein bands (white arrows) with molecular weights of more than 250 kDa. The band of 48 kDa (grey arrow) was also visible on a negative control blot. 

**Figure 4:**
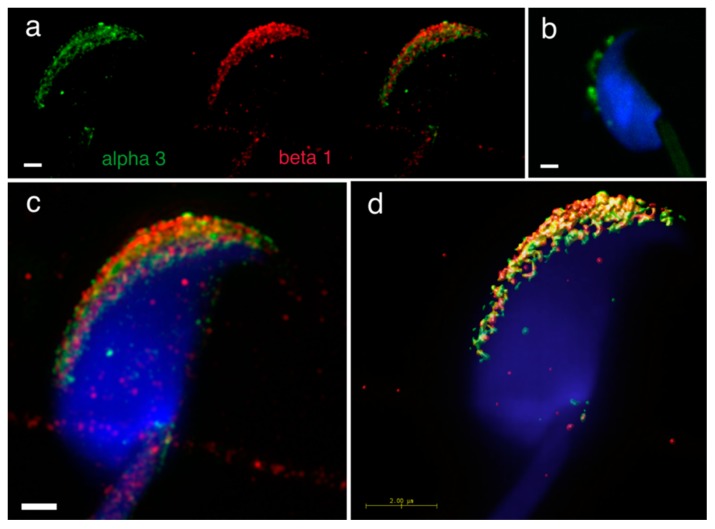
Mutual localization of α3 and β1 integrin subunit and a presence of α3β1 heterodimer revealed by SIM and PLA. (**a**) α3 (green) and β1 (red) are localized in the acrosomal cap area of intact sperm head, (**b**) PLA confirmed a presence of the α3β1 heterodimer. (**c**) SIM depicted their mutual localization in same structures. (**d**) Huygens software was used for better visualization of colocalization area (yellow) of α3 and β1. Colocalization maps are based on Pearson’s correlation coefficient. Nucleus is visualized with Dapi (blue). Scale bar represents 1 μm (**a**–**c**) and 2 μm (**d**).

Furthermore, we addressed heterodimer formation of the previously identified individual α3, α6 and β1 integrin subunits [24], as we were interested in defining integrin heterodimers in separate membrane compartments in the mouse sperm head. We used dual immunofluorescent staining visualized by 3D super resolution microscopy to visualize their localization by using Huygens software to generate a colocalization map based on Pearson’s correlation coefficient. The results confirmed the localization of α3 in the plasma membrane covering the acrosomal cap and in the outer acrosomal membrane (Figure 4a). In order to distinguish between membranes lying in close proximity, we used double immunofluorescent staining with CD46 as a marker of the acrosomal membrane [25] (Supplementary Figure S1). The localization of β1shared the same localization (Figure 4a), and their mutual interaction as α3β1 heterodimer was confirmed by PLA (Figure 4b). The share co-localization was confirmed by analysis of SIM data (Figure 4c) using Huygens software depicting a colocalization map based on Person’s coefficient (Figure 4d).

**Figure 5:**
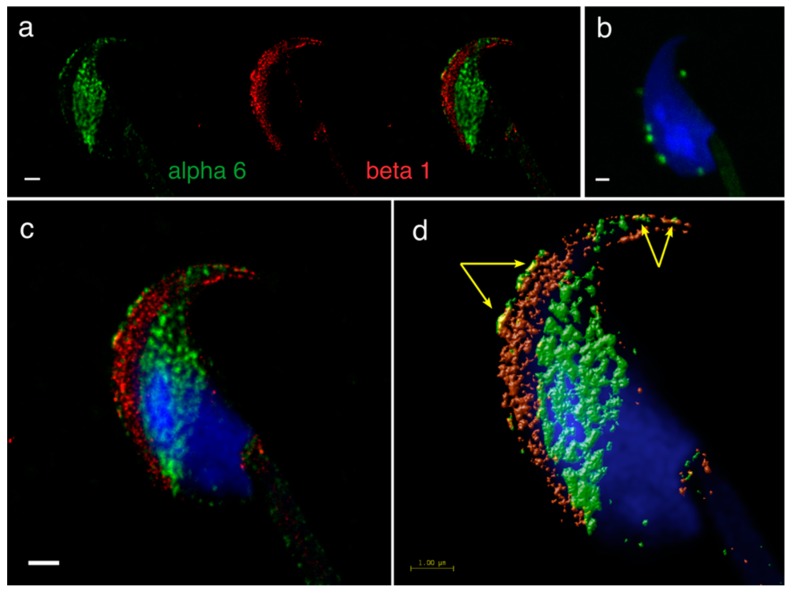
Mutual localization of α6 and β1 subunit and the presence of α6β1 heterodimer reveals by STED (Stimulated Emission Depletion) super-resolution microscopy and PLA. (**a**) α6 (green) is localized in plasma membrane over the acrosomal cap, apical hook and equatorial segment in contrast to β1 (red) localized in plasma membrane overlying the acrosomal cap stretching to apical hook and in outer acrosomal membrane. (**b**) PLA confirmed the presence of the α6β1 heterodimer in mutual places such as the plasma membrane over the acrosomal cap and apical hook. (**c**) STED dual-color imaging present overlay of both subunits. (**d**) Huygens software was used for better visualization of colocalization area (yellow, pointed by yellow arrows). Colocalization maps were based on Pearson’s correlation coefficient. Nucleus is visualized with Dapi (blue). Scale bar represents 1 μm (**a**–**d**).

In intact acrosome mouse sperm heads, the presence of the α6 subunit was shown in both the plasma membrane of acrosomal cap, apical hook and the equatorial segment, in contrast to the localization of β1, which was in the plasma membrane of the acrosomal cap area extending over the apical hook and in the outer acrosomal membrane (Figure 5a). PLA confirmed the presence of the α6β1 heterodimer specifically in the plasma membrane of the acrosomal cap area and the apical hook (Figure 5b) and a mutual colocalization of the α6 and β1 subunits was also visibly in the same locations (Figure 5c), which was confirmed by a colocalization map (Figure 5d, yellow arrows). 

**Figure 6:**
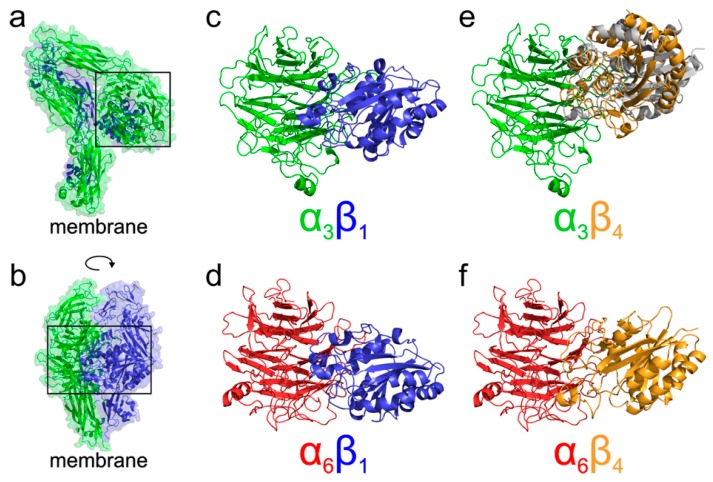
Docking of α/β integrin N-terminal domains. (**a**,**b**) An example of resting state structure of extracellular domains of the α3β1 integrin as obtained from I-TASSER homology modelling. As expected from the crystal structures available for threading by I-TASSER the overall architecture of the resulting model is similar to αXβ2 (PDB ID 4neh; [12]) or αIIbβ3 (PDB ID 3fcs; [13]) heterodimer structures. The interacting region of N-terminal domains is highlighted by the black rectangle. Panel (**a**) shows the α3 subunit on top of the β1 domains, while panel (**b**) represents the same structure rotated by 90 degrees orienting both the N-terminal domains to the front of the image. (**c**–**f**) The predicted arrangement of heterodimers formed by N-terminal domains of integrins α3β1 (**c**), α6β1 (**d**), α3β4 (**e**), and α6β4 (**f**) from the flexible side chain docking of the domains by ClusPro. In the case of α3β4 (**f**) two binding modes are shown. The predicted most stable dimer with the β4 domain (orange) interacting through the NV residues homologous with the typical Arg/Lys finger of β domains is compared to an alternative, less stable binding mode, employing the RPEK sequence (grey). Both binding modes are similar and suggest that the α3β4 complex adopts different domain orientation compared to the remaining α3β1, α6β1, and α6β4 cases. The α3 domains are shown in green, β1 in blue, α6 in red, and β4 in orange.

To better understand the different localization and behavior of the studied integrins, we performed molecular modeling of their four possible heterodimers (α3β1, α6β1, α3β4, and α6β4). Our analysis of existing crystal structures of integrin complexes in both the resting as well as activated open states agrees with the results of [7,13], showing that the majority of interactions are mediated by the N-terminal propeller domain of α integrins and βA (also called βI) domain of β integrins. The N-terminal domains model structures of integrins α3, α6, β1, and β4 were extracted from our homology models of the whole extracellular parts prepared employing the I-TASSER service [26] and further used for protein-protein docking analysis. The flexible side chain docking of N-terminal domains was performed using ClusPro server [27,28] to infer the most probable binding mode of various integrin combinations. Selected arrangements of N-terminal domains were subjected to a 100 ns long molecular dynamics simulation to identify possible artifacts of the docking. All the simulations showed that the domain orientations predicted by docking are stable throughout the simulation. The results of N-terminal domains docking are summarized in Figure 6, showing that α3β1, α6β1, and α6β4 complexes adopt the expected orientation of domains mediating the interaction with the Arg/Lys finger of β domains. On the other hand, in the case of α3β4 integrin, the docking suggested two binding modes. The predicted most stable dimer with the β4 domain interacting through the NV residues corresponding to the homologous Arg/Lys finger of remaining β domains is followed by a less stable interaction, employing the RPEK sequence. Both binding modes are similar and suggest that the α3β4 complex adopts different domain orientation compared to the remaining cases.

## 3. Discussion 

Laminin binding integrins α3β1, α6β1 and α6β4 are usually co-expressed in cells, and they are major receptors used by epithelial cells to bind laminins [29]. Interestingly, it has been recently shown that α3 and α6 subunits are able to cooperate and promote distinct function and signaling with the β1 subunit [30]. Even though all these integrin pairs interact with actin cytoskeleton, the heterodimer α6β4 is known to bind to actin and tubulin, but mainly to intermediate filament and provide mechanical and structural cell stability. 

**Figure 7:**
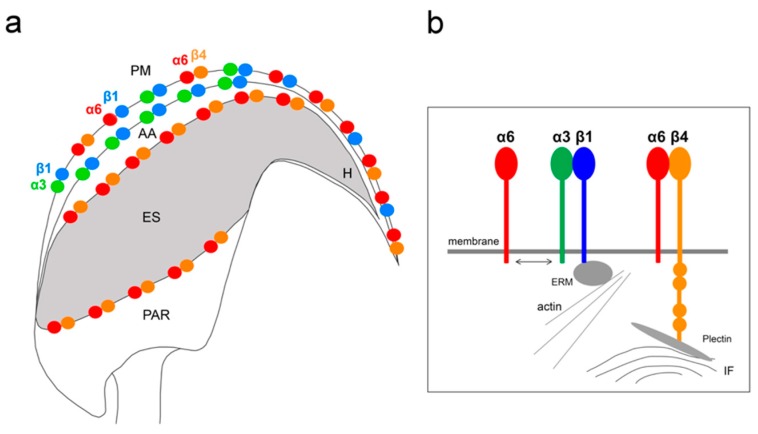
Graphical summary of integrin heterodimers and their localization in intact sperm head. (**a**) Localization of integrin heterodimers as follows: α3β1, α6β1, α6β4–plasma membrane (PM) overlaying the apical acrosome (AA), α6β1, α6β4–sperm hook (H); α3β–outer acrosomal membrane of apical acrosome (AA); α6β4–inner acrosomal membrane and equatorial segment (ES) up to posterior post-acrosomal region (PAR). (**b**) Schematic depiction of integrin subunits anchored in the membrane, dynamics between α3 and α6 with β1 and β subunits interaction with cytoskeleton.

In this study, we demonstrate the expression of the β4 integrin subunit in mouse testicular germ cells and mature spermatozoa, in which β4 occupies two distinct head compartments. Using super-resolution microscopy and PLA, we showed evidence that α6β4 exists in the mouse sperm head as a heterodimer, and is located in plasma membrane overlaying apical acrosome in a similar way to heterodimers α3β1 and α6β1; its location, together with α6β1, is also stretching over the sperm hook (Figure 7a). Moreover, we report on dimerization of β4 in the equatorial segment with the previously reported presence of the α6 integrin subunit in this sperm compartment [24] (Figure 7a). It may not be a coincidence that all three heterodimers share the same localization in the plasma membrane covering the distinct region of the acrosome. The plasma membrane is destined to fuse during the acrosome reaction with the outer acrosomal membrane, rich on α3β1, and the tight communication with the actin cytoskeleton is crucial for directing the ongoing event of acrosomal exocytosis [4,5]. In addition, due to a unique structure of the β4 cytoplasmic tail, which is able to interact with intermediate filaments such as keratin 5 via plectin [31,32,33] surrounding the nucleus, a largest organelle of the sperm may provide anchoring and stability (Figure 7b). The presence of the long β4 cytoplasmic tail in spermatozoa were proved by the q-RT-PCR and by sequencing of PCR products. Additionally, we found several further transcript variants, longer or shorter than those that are already known or predicted to exist. The specific antibody recognized a long protein variant (calculated molecular weight 204 kDa) and other higher protein isoforms. 

Based on the existing crystal structures combined with the docked homology models, we can further speculate about the functional consequences of the structural differences among α3β1, α6β1, α3β4, and α6β4 complexes. A model of probable arrangement of integrin heterodimers in the open state was derived from the results of N-terminal domains docking by a structure superposition of the existing crystal structure of αIIBβ3 in the open state (PDB ID 3fcu; [13]), see Figure 6 for a graphical summary. The modeling suggests that complexes of α3β1, α6β1, and α6β4 integrins are, as a consequence of the relative arrangement of their N-terminal domains, found in a conformation with the membrane proximal domains separated [34,35,36,37]. This leads to the expected cis interaction. On the other hand, the orientation of the α3β4 N-terminal domains would, however, translate to a complex with α and β subunits pointing in nearly opposite directions suggesting the trans interaction of the α3β4 integrin heterodimer. The preference for trans interaction in α3β4 integrin was further supported by an attempt to anchor the activated complex into the same membrane showing too strong a bending and separation of membrane proximal domains, which are necessary to form a complex (Supplementary Figure S2a). The model further supports the trans interaction by superposition of the resting integrin α3β4 subunits, leading also to the membrane proximal domains pointing in opposite directions, as summarized in the Supplementary Figure S2b.

**Figure 8:**
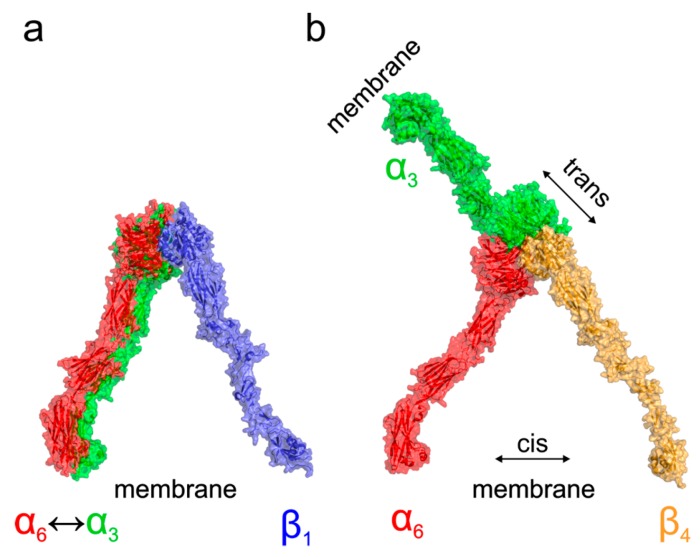
Model of activated states of α/β integrin heterodimers. A probable arrangement of integrin heterodimers in the open state was derived from the N-terminal domain complexes predicted by docking (see Figure 6) by a structural superposition of the existing crystal structure of αIIBβ3 in the open state (PDB ID 3fcu; [13]). The modelling suggests that while for the α3β1, α6β1 (**a**), and α6β4 (**b**) the integrins are involved in a cis interaction adopting the expected conformation with the membrane proximal domains separated [34,35,36,37], the orientation of the α3β4 N-terminal domains would lead to a complex with α and β subunits pointing in nearly opposite directions (**b**) supporting the possible trans interaction (see Supplementary Figure S2 for more details).

**Figure 9:**
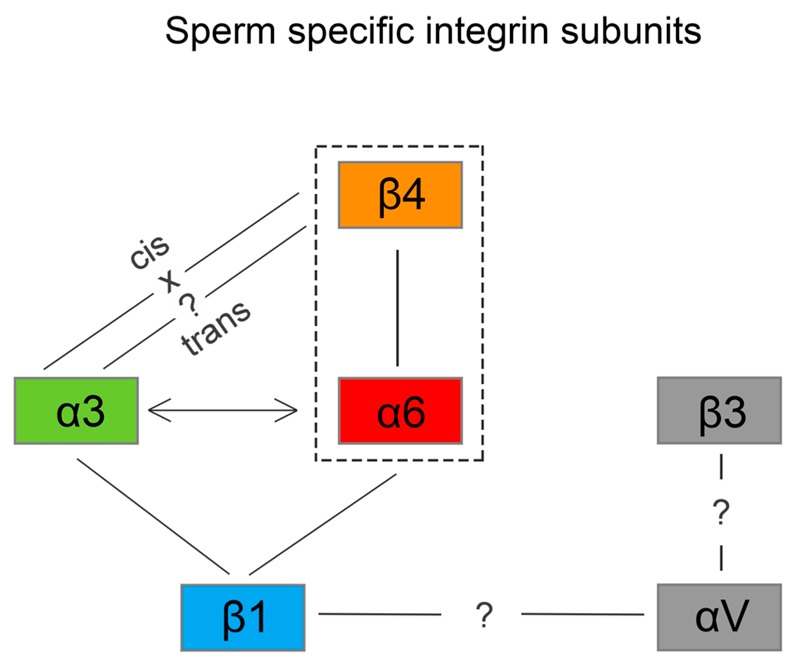
Diagram of sperm specific integrin subunits and heterodimers. The solid lines show proven integrin heterodimers α3β1, α6β1 and α6β4; the arrows show predicted α3 and α6 dynamics with β1; α3 and β4 cis interaction are energetically unstable in contract to trans interaction. Heterodimers between αV and β1/β3 are not known.

We also predict that during the acrosome reaction, when membrane fluidity increases, the α3 and α6 could interchange over the β1 subunit in a more flexible manner to modulate cytoskeletal function during membrane fusion (Figure 7 and Figure 8a). Moreover, active and inactive forms are known to occupy different clusters when only converting the inactive form into the active form, resulting in signaling activity. The membranes that become surface exposed after the acrosome exocytosis are also rich in integrins. However, to date, it seems that α3β1 and α6β4 integrins pairs are expressed on these sperm membranes which will be in first contact with the oolema (Figure 7). With knowledge that, among many others, α3 is also expressed on the egg surface [38], it may be of relevance that trans-interactions between α3 and β4 integrin subunits show favorable and stable trans position compared to the cis one (Figure 8b), for overall graphical interpretation of discussed interactions see Figure 9. The behavior that integrin-ligand binding activity happens in the extended active form; at the same time, both α and β cytoplasmic tails are not bound [37], which could facilitate the exchange of individual α subunits (Figure 9). 

The larger portion of integrins is usually found in the attached cells. With relevance to sperm-egg binding and membrane fusion, the existence of direct spatial organization of integrins into nanoclusters supports the coordinate mechanism of integrin activity regulation [39], which may be appropriate to consider during gamete interaction. 

Based on proteomic studies on subfertile men, α6β1 integrin has been proposed as a potential fertility marker for evaluating sperm quality [21]. We believe that understanding of physiology of integrins in sperm has a big potential in diagnostic medicine targeting idiopathic infertility in men. In the future, it will be of great importance to identify the integrin interaction with gamete tetraspanin network and classify molecules that could be conductors of the gamete recognition and sperm membrane reorganization. 

## 4. Materials and Methods 

### 4.1. Animals 

Inbred C57BL/6 mice were housed in a breeding colony of the Laboratory of Reproduction, IMG animal facilities, Institute of Molecular Genetics of Czech Academy of Science, and food and water were supplied ad libitum. The male mice used for all experiments were healthy, 10–12 weeks old, with no sign of stress or discomfort. All animal procedures and experimental protocols were approved by the AnimalWelfare Committee of the Czech Academy of Sciences (Animal Ethics Number 66866/2015-MZE-17214, 18 December 2015). 

### 4.2. Preparation of Testicular Cell Suspension

Two adult C57BL/6 mice were terminated and the testes were collected in RPMI medium (Sigma-Aldrich, Prague, Czech Republic). All testes deprived of tunica albuginea were placed in 10 ml RPMI medium containing 2.5 mg collagenase (Sigma-Aldrich, C7657) and 0.5 mg Dnase I (Roche, Basel, Switzerland) and agitated at 33 °C, 50 rpm for 20 min. Then, the testes were gently pipetted 10–20× to help separation of seminiferous tubules from interstitial tissue and filtrated through a 40 µm cell strainer. Seminiferous tubules were transferred into 10 mL RPMI medium containing 5 mg collagenase (Sigma-Aldrich, C7657) and 0.5 mg Dnase I (Roche) and agitated at 33 °C, 50 rpm for next 20 min. After incubation, the tubules were pipetted 10–20× to help separation of individual cells. Single cell suspension was filtrated through a 70 µm following 40 µm cell strainer to remove cell clumps. Then, the suspension was centrifuged at 400 g, 4 °C for 10 min. The pellet was resuspended in fresh RPMI medium (4 °C) containing Dnase I (Roche) and kept on ice until further analysis.

### 4.3. Elutriation

Centrifuge J26XP with elutriation rotor JE-5.0 (Beckman Coulter, Indianapolis, IN, USA) were used for elutriation. Elutriation protocol was performed as previously described [40] with small modifications. The elutriation protocol was done in PBS at 4 °C; the precise conditions are described in Supplementary Table S2. The cells were collected into 50 mL tubes that were kept on ice. Cells in each tube were pelleted by centrifugation (400 g, 15 min, 4 °C) and resuspended in Tri-reagent (Sigma-Aldrich). The total RNA was isolated according to manufacturer’s instructions and stored at −70 °C.

### 4.4. RNA Isolation

Total RNA was isolated from testicular fractions prepared by elutriation and testes samples using TRI Reagent (Sigma-Aldrich). The concentration and quality of RNA were measured using Nanodrop spectrophotometer Helios α (Thermo Scientific, Waltham, MA, USA).

### 4.5. Reverse Transcription (cDNA Synthesis)

The volume of each RNA extract was adjusted to 2 μg for each template. Firstly, RNA extracts were treated with DNase I (1 U/μL, Fermentas, Hanover, MA, USA) in presence of DNase I buffer 10× (Thermo Scientific) with MgCl_2_ for 30 min at 37 °C. Subsequently, EDTA (Fermentas) was added for 10 min at 65 °C. The reverse transcription reaction contained 5× reaction Buffer (Fermentas), Riboblock Inhibitor (20 U/μL, Sigma-Aldrich), Universal RNA Spike II (0.005 ng/μL, TATAA biocenter, Göteborg, Sweden), 10 mM dNTP Mix (Thermo Scientific), oligo(dt)18 (Thermo Scientific) mixed 1:1 with Random primers (Thermo Scientific) and M-MuLV RevertAid transcriptase (200 U/μL, Fermentas), and run for 60 min at 42 °C followed by 10 min at 70 °C to generate cDNA. Obtained cDNA was stored at −20 °C. All cDNA samples were synthesized in duplicates. Negative control RT^−^ was prepared in the same conditions but with RNase/DNase free water (Thermo Scientific) instead of the Reverse transcriptase.

### 4.6. Real Time Quantitative PCR (q-RT-PCR)

For q-RT-PCR, 10× diluted cDNA (10 ng/µl) was used. Two times Maxima SYBR Green qPCR Master Mix (Thermo Scientific), reverse and forward primer (1 μM, Generi Biotech, Hradec Kralove, Czech Republic) and nuclease free water were used. q-RT-PCR reaction was carried out in 384-PCR well plates (Bio-Rad, Hercules, CA, USA), and all reactions were performed in duplets in a PCR cycler (CFX 384–qPCR cycler, Bio-Rad). 

The *Ribosomal protein S2* (*Rps2*) gene was used as the reference gene. Specific gene markers for germinal cells and somatic cells were used to determine elutriation fractions. The mRNA expression of target genes was calculated based on the quantification cycle (Cq) difference (Δ) of a testicular elutriation fractions versus testis.

Primers above 97% qPCR efficiency with one specific melting peak were used for the analysis (primer sequences are listed in the Supplementary Table S3). Negative control (NTC) was prepared in the same conditions, except that cDNA was replaced by nuclease free water. RT^−^ negative control for cDNA synthesis was also analyzed. 

### 4.7. DNA Agarose Electrophoresis and Sequencing PCR Products of β4 Integrin Subunit

The 2% agarose gel was prepared with adding Gel red staining (Biotium, Fremont, CA, USA) was done following Zymoclean Gel DNA Recovery kit instruction (Zymo Research, Irvine, CA, USA). DNA fragments were excised from agarose gel. Agarose gel with DNA fragments were dissolved in agarose dissolved buffer at 55 °C for 10 min. The melted agarose solution was transferred to a Zymo-Spin Column in a collection tube and centrifuged for 60 s. Volume of 200 μL was added and washing step was repeated. Finally, the column matrix was eluted in DNA Elution Buffer and centrifuged for 60 s. The final concentration was measured in NanoDrop spectrophotometer Helios α (Thermo Scientific). The sequencing of the selected PCR products was done by a commercial company (SEQme, Prague, Czech Republic).

### 4.8. SDS-PAGE Electrophoresis and Western Blotting

Sodium dodecyl sulphate-polyacrylamide gel electrophoresis (SDS-PAGE) was carried out in 10% slab gel as described by Laemmli [41]. Proteins from mice epididymal sperm were extracted by 1% Triton X-100 in 50 mM Tris-HCl (pH 7.8) with 30 mM KCl and protease inhibitors (Roche Diagnostics, Mannheim, Germany). Extracted proteins were precipitated by frozen acetone and dissolved in non-reducing buffer and boiled for 5 min at 100 °C. The molecular masses of the separated proteins were estimated using pre-stained precision protein standards Dual color (Bio-Rad). Tris–glycine buffer (pH 9.6) with 20% methanol was used for the transfer of proteins separated by SDS-PAGE onto the PVDF membrane Immobilon-P (Millipore, Darmstadt, Germany) for immunodetection. Electroblotting was carried out for 2 hours at 500 mA.

### 4.9. Protein Immunodetection

The PVDF membrane with the transferred proteins was deactivated with 5% dry milk (Bio-Rad) in PBS at 1 h in room temperature. After washing with 0.1% Tween 20 in PBS, the membrane was incubated with mouse monoclonal anti-β4 integrin (sc-13543, Santa Cruz Biotechnology, Inc., Dallas, TX, USA) 1:1000 diluted in PBS overnight at 4 °C. Following washing, incubation with anti-mouse immunoglobulins coupled to horseradish peroxidase (Bio-Rad) diluted 1:3000 in PBS was performed for 1 h at room temperature. After washing, the membrane was developed with SuperSignal Chemiluminescent Substrate (Thermo Scientific). As a negative control, the membrane was incubated with mouse IgG (0.1 µg/mL; Sigma-Aldrich) and anti-mouse secondary antibody.

### 4.10. Immunofluorescent Detection of Integrin Subunits with Confocal and Super-Resolution Microscopy (SRM)

Freshly released epididymal sperm were used for confocal microscopy and SIM and STED super-resolution microscopy. Sperm from the distal regions of cauda epididymis were released into a 200 μL droplet of M2-fertilising medium (Sigma-Aldrich, M7167) under paraffin oil (P-Lab, Prague, Czech Republic, P14501) in a Petri dish and pre-tempered at 37 °C in the presence of 5% CO_2_. Released sperm were assessed for motility and viability under a light inverted microscope with a thermostatically controlled stage at 37 °C. 

Sperm were washed twice in PBS, smeared onto glass slides and air-dried. For SRM sperm samples were always prepared onto high precision cover glasses (thickness No. 1.5 H, 170 ± 5 µm, Marienfeld, Germany). Sperm smears were fixed with 3.7% formaldehyde in PBS (pH 7.34) (in case of α3β1 and α6β1 heterodimer) at room temperature for 10 min or with acetone at −20 °C for 10 min (in case of α6β4 heterodimer), followed by washing in PBS. Sperm were blocked with 10% BSA in PBS for 1 h and incubated with primary rabbit polyclonal antibody anti-β1 integrin (sc-8978, Santa Cruz Biotechnology, Inc.) diluted 1:10 in PBS and/or primary goat polyclonal antibody anti-α3 integrin (N19) (sc-6588, Santa Cruz Biotechnology, Inc., Dallas, TX, USA) 1:10, mouse monoclonal antibody anti α6 integrin (F6) (sc-374057, Santa Cruz Biotechnology, Inc.) 1:10, rabbit polyclonal antibody anti-β4 integrin (bs-4115R, Bioss antibodies, Woburn, MA, USA) 1:10 in PBS over night at 4 °C, followed by Alexa Fluor 488 goat anti-rabbit IgG and/or Alexa Fluor 568 donkey anti-goat IgG (Molecular Probes, Eugene, OR, USA), Alexa Fluor 568 donkey anti-mouse IgG (Molecular Probes) secondary antibodies 1:300 in PBS for 1 h at room temperature. In case of dual staining, both primary or secondary antibodies were applied together. After the application of the primary and secondary antibodies, sperm were incubated for 5 min with DAPI (0.85 µg/mL, Thermo Scientific, Waltham, MA, USA) and washed 3× in PBS. At the end, sperm were washed 1× in distilled water and air-dried. Dry samples were covered with 90% glycerol with 5% anti-fade N-propyl gallate (Sigma-Aldrich). Multi-color SIM super-resolution images were obtained by Zeiss Elyra PS.1 inverted microscope at Laboratory of confocal and fluorescent microscopy of Faculty of Science (Charles University, Prague, Czech Republic). STED images were obtained by Leica TCS SP8 STED 3X microscope (Microscopy centre—LM and EM, IMG AS, Prague, Czech Republic). Confocal data for colocalization analysis of α6 and β4 subunit was collected with high-end confocal microscope Carl Zeiss LSM 880 NLO (Imaging Methods Core Facility at BIOCEV, Vestec, Czech Republic). An open source software Fiji [42] was used for further image processing.

### 4.11. Proximity Ligation Assay

To detect the protein-protein interaction of α3β1, α6β1, α6β4 integrins, Proximity Ligation Assay Duolink (PLA) was used. The principle of the method, and possibilities of its use for confirmation of protein-protein interactions, was described by Söderberg et al. [43]. Proteins α tubulin (DM1A, Sigma, 1:20) and β tubulin (sc-9104, Santa Cruz Biotechnology, Inc., 1:10) were selected as a positive control (DUO92101 Duolink, In Situ Red Starter Kit Mouse/Rabbit, Olink Bioscience), β1 integrin (sc-8978, Santa Cruz Biotechnology, Inc.) and α tubulin (DM1A, Sigma) as a negative control (DUO92101 Duolink. In Situ Red Starter Kit Mouse/Rabbit, Olink Bioscience). The interaction of experimental proteins, α6β1, α6β4 was studied using a DUO92101 Duolink. In Situ Red Starter Kit Mouse/Rabbit, Olink Bioscience and α3β1 DUO92006-30RXN, Duolink. In Situ Red Starter Kit Mouse/Goat, Olink Bioscience. Freshly released sperm were washed twice in PBS, smeared onto glass slides and air-dried. Sperm smears were fixed with 3.7% formaldehyde in PBS (pH 7.34) at room temperature for 10 min, followed by washing in PBS. For α6β4 was used fixation in acetone for 10 min in −20 °C. Sperm were blocked with 10% BSA in PBS for 1 h and incubated with primary antibodies. In each experiment, two primary antibodies were used, each directed against one of the target proteins. These antibodies were raised in different species. Species-specific secondary antibodies (PLA probes) bind to primary antibodies, and each of them has a unique short DNA strand attached to it. Both DNA strands interacted through a subsequent addition of two other circle-forming DNA oligonucleotides, forming a DNA circle, which was closed by DNA Ligation. DNA circles were amplified using a DNA polymerase. The amplified DNA was detected by hybridization with labeled oligonucleotides, which produced a visible fluorescent spot. These spots were detected with high-end confocal microscope Carl Zeiss LSM 880 NLO (Imaging Methods Core Facility at BIOCEV, Vestec, Czech Republic). Representative results are shown.

### 4.12. Molecular Modelling of Integrin Heterodimers

The all atom models of extracellular parts of integrins α3, α6, β1, and β4 were prepared using a local copy of the I-TASSER [26] service, based on the annotation of protein sequences as defined by uniprot accession codes Q62470, Q61739, P09055, and P09055. The flexible side chain protein-protein docking of N-terminal domains was performed using a local copy of the ClusPro server [27,28]. The GBSA implicit solvation molecular dynamics simulations were prepared using the OpenMM Zephyr graphical interface [44] with the Amber96 force field, 2 fs time step, temperature 295 K, and water collisional interval of 0.01099 ps. The calculations were performed using the GPU accelerated version of GROMACS program [45] collecting geometry every 10 ps. Analysis of modelling results, structure superposition and graphical visualization was performed using the PYMOL program [46] version 2.1.0.

### 4.13. Data Analysis

Huygens Professional version 18.10 (Scientific Volume Imaging, Hilversum, The Netherlands, Available online: http://svi.nl) was used for visualization mutual position of individual proteins based on surface rendering of the colocalization analysis. A colocalization analyzer computed a Pearson’s correlation coefficient and created a 3D colocalization map. The Pearson’s correlation coefficient expresses the rate of correlation of colocalizing channels in a dual-color image, giving a value os between minus 1 and plus 1. In this case, 1 means an absolutely positive correlation, 0 means no correlation and −1 means a perfect anti-correlation. The value between 0.5 and 1 is interpreted as colocalization. The Costes method was used for a background estimation. Ten individual sperm confocal images were analyzed by Fiji software using customized JACoP (Just Another Colocalization Plugin) plugin to calculate Pearson’s coefficients [47], and the resulting values were statistically evaluated to determine the arithmetic mean and standard deviation. 

## 5. Conclusions

In conclusion, we delivered a detailed characterization of α3, α6, β1 and β4 integrin subunits showing their presence in distinct compartments of the intact mouse sperm head. Moreover, we identified sperm-specific localization for heterodimers α6β4, α3β1 and α6β1, and their membrane compartmentalization and the presented data show a complexity of membranes overlaying specialized microdomain structures in the sperm head. Their different protein composition may correspond with the specialized role of these individual membrane rafts, facilitating in and out signaling events during sperm maturation, acrosome reaction, as well as sperm-epithelium and sperm-egg interaction. 

## Figures and Tables

**Table 1:** Gene expression of β4 integrin in the cell-type fractions. Cq value of the gene is normalized by reference gene *Rps2*. Numbers > 1 are considered as strongly expressed in the individual cell-types.

Target Gene	Testicular Elutriation Fractions					
	Round Spermatids	Round Spermatids	Spermatogonia	Spermatogonia	Primary spermatocytes	Testes
*Itg β4*	1.845	5.276	10.623	13.368	2.637	1

## Supplementary Materials

Supplementary materials can be found at https://www.mdpi.com/1422-0067/20/5/1004/s1.
