# Supplementary material for: Addressing the Compartmentalization of Specific Integrin Heterodimers in Mouse Sperm
Source: Int J Mol Sci. 2019 Feb 26;20(5):1004. doi: 10.3390/ijms20051004 (PMC6429177; doi:10.3390/ijms20051004)
Supplement: Supplementary file 1 [file ijms-20-01004-s001.zip › Frolikova_Supplementary.docx]

**Addressing the compartmentalization of specific integrin heterodimers in mouse sperm**

Michaela Frolikova^1^, Eliska Valaskova^1^, Jiri Cerny^3^, Audrey Lumeau^1,2^, Natasa Sebkova^1^, Veronika Palenikova^1,4^, Noemi Sanches-Hernandez^1^, Alzbeta Pohlova^1,4^, Pavla Manaskova-Postlerova^1,5^ and Katerina Dvorakova-Hortova^1,6*^

*^1^Laboratory of Reproductive Biology, Institute of Biotechnology CAS, v.v.i., BIOCEV, Prumyslova 595, 252 50, Vestec, Czech Republic.*

*^2^Group of Cell Biology and Innovative Therapies, Centre for Molecular Biophysics, University of Orleans, UPR4301, Orleans, France.*

*^3^Laboratory of Structural Bioinformatics of Proteins, Institute of Biotechnology CAS, v.v.i., BIOCEV, Prumyslova 595, 252 50, Vestec, Czech Republic.*

*^4^Department of Biochemistry, Faculty of Science, Charles University, Vinicna 7, Prague 2, 128 44, Czech Republic.*

*^5^Department of Veterinary Sciences, Faculty of Agrobiology, Food and Natural Resources, University of Life Sciences Prague, Kamycka 129, Prague 6, 165 00, Czech Republic.*

*^6^Department of Zoology, Faculty of Science, Charles University, Vinicna 7, Prague 2, 128 44, Czech Republic.*

**Supplementary figures and tables**


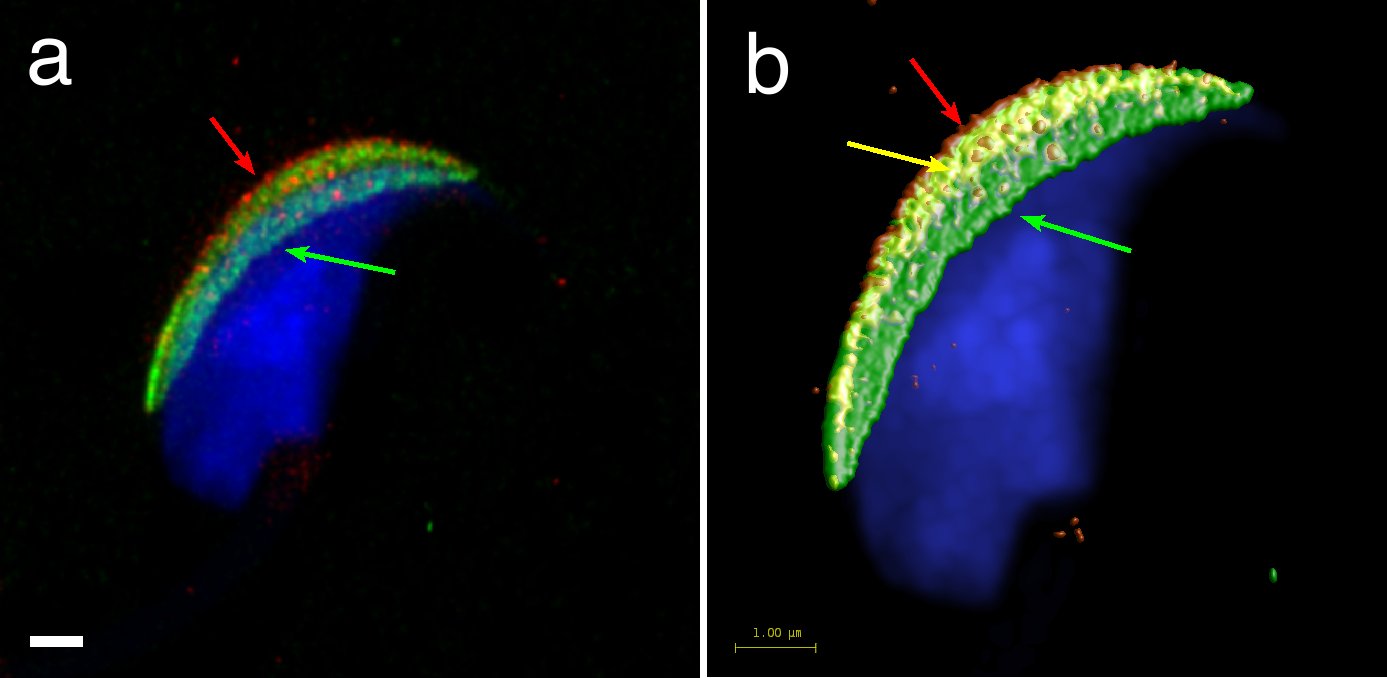


**Supplementary Figure 1. Pinpointing the localization of α3 in specific mouse sperm head membrane by STED super-resolution microscopy.** (a) STED data showed localization of α3 (red) in the plasma membrane of the acrosomal cap area (see red arrow in a and b) and in the outer acrosomal membrane (yellow in b) of intact mouse sperm head. Protein CD46 was used as a marker of both compartments of the acrosomal membrane and only green signal was detected in the inner acrosomal membrane (see green arrow in a and b). (b) Co-localization analysis and surface rendering was used with Huygens software for better visualization of localization of α3. A co-localization map based on Pearson’s correlation coefficient showed α3 localization in the outer acrosomal membrane (see yellow arrow). Nucleus is visualized with Dapi (blue). Scale bar represents 1 µm (a, b).

**
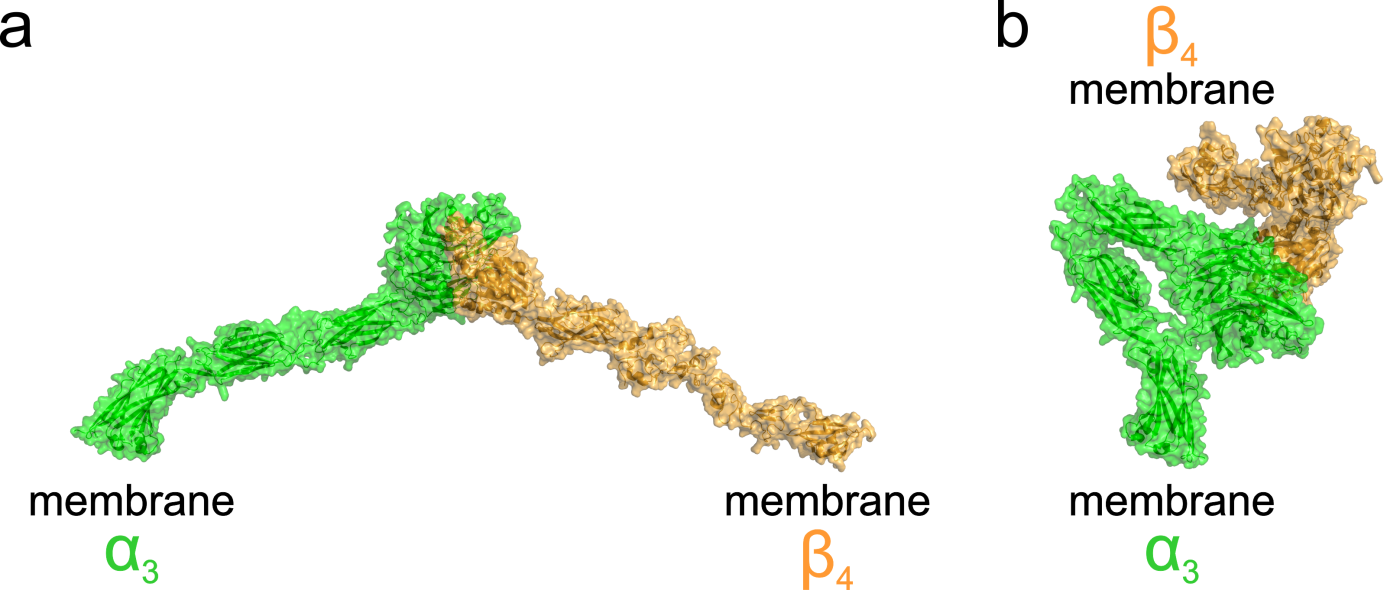
**

**Supplementary Figure 2. Preferential trans over cis interaction for the integrin α3β4.** The suggested preference for trans interaction in α3β4 integrin as shown in Fig 9 is further supported by an attempt to anchor the activated complex into the same membrane **(a)** showing too strong bending and separation of membrane proximal domains necessary to form a complex. The data in panel **(b)** further support the trans interaction by superposition of the resting integrin α3β4 subunits leading also to the membrane proximal domains pointing in opposite directions.

**Supplementary Table 1.** Characterization of the cell population of testicular elutriation fractions using specific gene markers for each population of cells by RT-qPCR. Normalization by *Rps2* housekeeping gene. Data show relativity between cell populations and whole testes; more than 1 is consider as really well enriched by the cell-type.

| **Gene markers** | **Theoretical cell populations after elutriation** | | | | | | **Primary source** |
| --- | --- | --- | --- | --- | --- | --- | --- |
|  | Fraction 1 | Fraction 2 | Fraction 3 | Fraction 4 | Fraction 5 | testes |  |
| *C-kit* | 0.399 | 1.325 | **3.807** | **4.306** | 0.677 | 1 | Spermatogonia |
| *Sycp3* | 0.356 | 0.903 | 1.286 | 1.665 | **1.029** | 1 | Primary spermatocytes |
| *Acrv1* | **1.520** | **2.244** | 1.300 | 0.571 | 0.309 | 1 | Round spermatids |
| *Dbil5* | 0.943 | 0.975 | 0.447 | 0.239 | 0.202 | 1 | Round/elongating spermatids |
| *Cyp11a1* | 0.008 | 0.083 | 0.838 | 2.452 | 0.858 | 1 | Leydig cells |
| *Wt1* | 0.062 | 0.221 | 0.224 | 0.219 | 0.131 | 1 | Sertoli cells |

**Supplementary Table 2.** Elutriation conditions.

|  | Rotor speed (rpm) | flow rate (ml/min) | volume collected (ml) |
| --- | --- | --- | --- |
| Washing | 1000 | 10 |  |
| Loading chamber | 2000 | 10 |  |
| Fraction 1 | 2000 | 12 | 100 |
| Fraction 2 | 2000 | 15 | 100 |
| Fraction 3 | 2000 | 25 | 100 |
| Fraction 4 | 2000 | 30 | 100 |
| Fraction 5 | 2250 | 37 | 100 |

**Supplementary Table 3.** Primer sequences of elutriation gene markers, reference gene and β4 integrin.

| **Gene symbol** | **Gene name** | **RefSeq ID** | **Primer sequence** | |
| --- | --- | --- | --- | --- |
| *C-kit* | Tyrosine kinese protein kit or CD117 | NM_001122733.1 | forward | 5' -CGTCTTCCGGCACAACGGCA- 3' |
|  |  |  | reverse | 5' -AGCAGCGGCGTGAACAGAGTG- 3' |
| *Sycp3* | Synaptonemal Complex Protein 3 | NM_011517.2 | forward | 5' -GGACAGCGACAGCTCACCGG- 3' |
|  |  |  | reverse | 5' -GGTGGCTTCCCAGATTTCCCAGA- 3' |
| *Acrv1* | Activin A receptor, type I | Yao-Fu Chang et al., 2011 | forward | 5' -TCAGCAACTTTCAAGCGAGTAT-3' |
|  |  |  | reverse | 5' -CTCCTGAAGAGTGCTCACCTG- 3' |
| *Dbil5* | Diazepam-binding inhibitor-like 5 | Yao-Fu Chang et al., 2011 | forward | 5'- CCCAGGGCGACTGTAACATC- 3' |
|  |  |  | reverse | 5' -GCAATGTAGATCCTCATGGCAT- 3' |
| *Cyp11a1* | Cytochrome P450 family 11 subfamily A member 1 | Yao-Fu Chang et al., 2011 | forward  reverse | 5' -CCAGTGTCCCCATGCTCAAC- 3'  5' -TGCATGGTCCTTCCAGGTCT- 3' |
| *Wt1* | Wilms Tumor 1 |  | forward | 5' -GGCGCTTTGAGGGGTCCGAC- 3' |
|  |  |  | reverse | 5' -AAAGTGGGCGGAGCACCGAC- 3' |
| *Rps2* | Ribosomal protein S2 | Yao-Fu Chang et al., 2011 | forward | 5' -CTGACTCCCGACCTCTGGAAA- 3' |
|  |  |  | reverse | 5' -GAGCCTGGGTCCTCTGAACA- 3' |
| *Itg β4* | β4 integrin | NM_001005608.2 | forward | 5' -AAGTCCAACTCAGCAACCCC- 3' |
|  |  |  | reverse | 5' -AGACTCCTGTCCGTTTCATCG- 3' |

| primer pair 1 | Cytoplasmic domain of Itg β4 | NM_001005608.2 | forward | 5' -GGATGAGGATGACGACTGCA- 3' |
| --- | --- | --- | --- | --- |
|  |  |  | reverse | 5' -TATTCAGGCTGCTCGAAGGA- 3' |
| primer pair 2 | Cytoplasmic domain of Itg β4 | NM_001005608.2 | forward | 5' -AGGCCATTGATGTCCCTGTG- 3' |
|  |  |  | reverse | 5' -ATGGGTCGGTTGTCCTCATT- 3' |
| primer pair 3 | Cytoplasmic domain of Itg β4 | NM_001005608.2 | forward | 5' -GCCCTATAGCTCACTGGTGT- 3' |
|  |  |  | reverse | 5' -CTGCCAGCTCACCTTCAAAG- 3' |
| primer pair 4 | Cytoplasmic domain of Itg β4 | NM_001005608.2 | forward | 5' -ACTCTATAATCCTGGCCGGG- 3' |
|  |  |  | reverse | 5' -TCACGAATTCCTGGGTCACA- 3' |
| primer pair 5 | Cytoplasmic domain of Itg β4 | NM_001005608.2 | forward | 5' -TCTTCCAGAACCCAGTGCAA- 3' |
|  |  |  | reverse | 5' -GGTGGGATGCAGTCAGAAAG- 3' |

**References**

Chang, Y. F.; Lee-Chang, J.S.; Panneerdoss, S.; MacLean, J. A. 2nd & Rao, M. K. Isolation of Sertoli, Leydig, and spermatogenic cells from the mouse testis. *Biotechniques*. **2011**, 51(5), 341-342, 344. doi: 10.2144/000113764.
