# Supplementary material for: Addressing the Compartmentalization of Specific Integrin Heterodimers in Mouse Sperm
Source: Int J Mol Sci. 2019 Feb 26;20(5):1004. doi: 10.3390/ijms20051004 (PMC6429177; doi:10.3390/ijms20051004)
Supplement: Supplementary file 1 [file ijms-20-01004-s001.zip › Supplementary Table 1.docx]

**Supplementary Table 1.** Characterization of the cell population of testicular elutriation fractions using specific gene markers for each population of cells by RT-qPCR. Normalization by *Rps2* housekeeping gene. Data show relativity between cell populations and whole testes; more than 1 is consider as really well enriched by the cell-type.

| **Gene markers** | **Theoretical cell populations after elutriation** | | | | | | **Primary source** |
| --- | --- | --- | --- | --- | --- | --- | --- |
|  | Fraction 1 | Fraction 2 | Fraction 3 | Fraction 4 | Fraction 5 | testes |  |
| *C-kit* | 0.399 | 1.325 | **3.807** | **4.306** | 0.677 | 1 | Spermatogonia |
| *Sycp3* | 0.356 | 0.903 | 1.286 | 1.665 | **1.029** | 1 | Primary spermatocytes |
| *Acrv1* | **1.520** | **2.244** | 1.300 | 0.571 | 0.309 | 1 | Round spermatids |
| *Dbil5* | 0.943 | 0.975 | 0.447 | 0.239 | 0.202 | 1 | Round/elongating spermatids |
| *Cyp11a1* | 0.008 | 0.083 | 0.838 | 2.452 | 0.858 | 1 | Leydig cells |
| *Wt1* | 0.062 | 0.221 | 0.224 | 0.219 | 0.131 | 1 | Sertoli cells |
