# Supplementary material for: Addressing the Compartmentalization of Specific Integrin Heterodimers in Mouse Sperm
Source: Int J Mol Sci. 2019 Feb 26;20(5):1004. doi: 10.3390/ijms20051004 (PMC6429177; doi:10.3390/ijms20051004)
Supplement: Supplementary file 1 [file ijms-20-01004-s001.zip › Supplementary Table 2.docx]

**Supplementary Table 2.** Elutriation conditions.

|  | Rotor speed (rpm) | flow rate (ml/min) | volume collected (ml) |
| --- | --- | --- | --- |
| Washing | 1000 | 10 |  |
| Loading chamber | 2000 | 10 |  |
| Fraction 1 | 2000 | 12 | 100 |
| Fraction 2 | 2000 | 15 | 100 |
| Fraction 3 | 2000 | 25 | 100 |
| Fraction 4 | 2000 | 30 | 100 |
| Fraction 5 | 2250 | 37 | 100 |
