# Supplementary material for: Addressing the Compartmentalization of Specific Integrin Heterodimers in Mouse Sperm
Source: Int J Mol Sci. 2019 Feb 26;20(5):1004. doi: 10.3390/ijms20051004 (PMC6429177; doi:10.3390/ijms20051004)
Supplement: Supplementary file 1 [file ijms-20-01004-s001.zip › Supplementary Table 3.docx]

**Supplementary Table 3.** Primer sequences of elutriation gene markers, reference gene and β4 integrin.

| **Gene symbol** | **Gene name** | **RefSeq ID** | **Primer sequence** | |
| --- | --- | --- | --- | --- |
| *C-kit* | Tyrosine kinese protein kit or CD117 | NM_001122733.1 | forward | 5' -CGTCTTCCGGCACAACGGCA- 3' |
|  |  |  | reverse | 5' -AGCAGCGGCGTGAACAGAGTG- 3' |
| *Sycp3* | Synaptonemal Complex Protein 3 | NM_011517.2 | forward | 5' -GGACAGCGACAGCTCACCGG- 3' |
|  |  |  | reverse | 5' -GGTGGCTTCCCAGATTTCCCAGA- 3' |
| *Acrv1* | Activin A receptor, type I | Yao-Fu Chang et al., 2011 | forward | 5' -TCAGCAACTTTCAAGCGAGTAT-3' |
|  |  |  | reverse | 5' -CTCCTGAAGAGTGCTCACCTG- 3' |
| *Dbil5* | Diazepam-binding inhibitor-like 5 | Yao-Fu Chang et al., 2011 | forward | 5'- CCCAGGGCGACTGTAACATC- 3' |
|  |  |  | reverse | 5' -GCAATGTAGATCCTCATGGCAT- 3' |
| *Cyp11a1* | Cytochrome P450 family 11 subfamily A member 1 | Yao-Fu Chang et al., 2011 | forward  reverse | 5' -CCAGTGTCCCCATGCTCAAC- 3'  5' -TGCATGGTCCTTCCAGGTCT- 3' |
| *Wt1* | Wilms Tumor 1 |  | forward | 5' -GGCGCTTTGAGGGGTCCGAC- 3' |
|  |  |  | reverse | 5' -AAAGTGGGCGGAGCACCGAC- 3' |
| *Rps2* | Ribosomal protein S2 | Yao-Fu Chang et al., 2011 | forward | 5' -CTGACTCCCGACCTCTGGAAA- 3' |
|  |  |  | reverse | 5' -GAGCCTGGGTCCTCTGAACA- 3' |
| *Itg β4* | β4 integrin | NM_001005608.2 | forward | 5' -AAGTCCAACTCAGCAACCCC- 3' |
|  |  |  | reverse | 5' -AGACTCCTGTCCGTTTCATCG- 3' |

| primer pair 1 | Cytoplasmic domain of Itg β4 | NM_001005608.2 | forward | 5' -GGATGAGGATGACGACTGCA- 3' |
| --- | --- | --- | --- | --- |
|  |  |  | reverse | 5' -TATTCAGGCTGCTCGAAGGA- 3' |
| primer pair 2 | Cytoplasmic domain of Itg β4 | NM_001005608.2 | forward | 5' -AGGCCATTGATGTCCCTGTG- 3' |
|  |  |  | reverse | 5' -ATGGGTCGGTTGTCCTCATT- 3' |
| primer pair 3 | Cytoplasmic domain of Itg β4 | NM_001005608.2 | forward | 5' -GCCCTATAGCTCACTGGTGT- 3' |
|  |  |  | reverse | 5' -CTGCCAGCTCACCTTCAAAG- 3' |
| primer pair 4 | Cytoplasmic domain of Itg β4 | NM_001005608.2 | forward | 5' -ACTCTATAATCCTGGCCGGG- 3' |
|  |  |  | reverse | 5' -TCACGAATTCCTGGGTCACA- 3' |
| primer pair 5 | Cytoplasmic domain of Itg β4 | NM_001005608.2 | forward | 5' -TCTTCCAGAACCCAGTGCAA- 3' |
|  |  |  | reverse | 5' -GGTGGGATGCAGTCAGAAAG- 3' |

**References**

Chang, Y. F.; Lee-Chang, J.S.; Panneerdoss, S.; MacLean, J. A. 2nd & Rao, M. K. Isolation of Sertoli, Leydig, and spermatogenic cells from the mouse testis. *Biotechniques*. **2011**, 51(5), 341-342, 344. doi: 10.2144/000113764.
