# Supplementary figures and images for: Addressing the Compartmentalization of Specific Integrin Heterodimers in Mouse Sperm
Source: Int J Mol Sci. 2019 Feb 26;20(5):1004. doi: 10.3390/ijms20051004 (PMC6429177; doi:10.3390/ijms20051004)

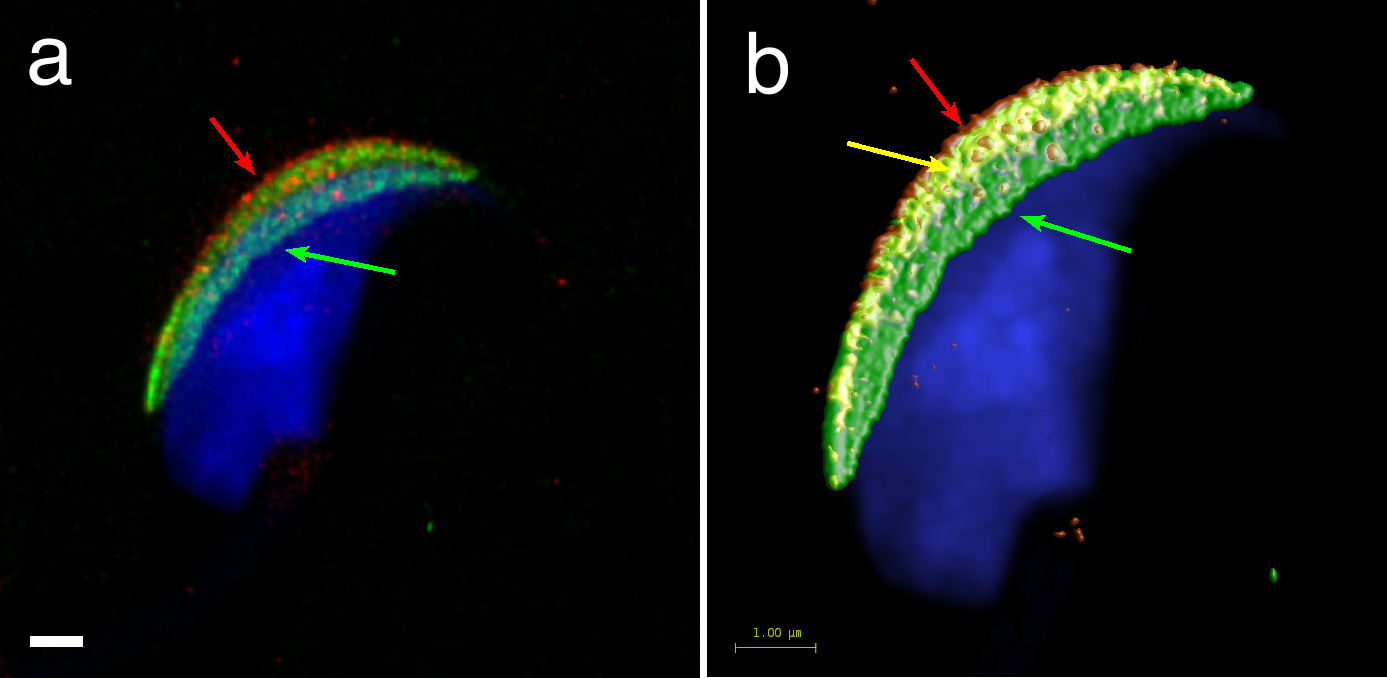

Supplement: Supplementary file 1 [file ijms-20-01004-s001.zip › Supplementary Fig 1.png]

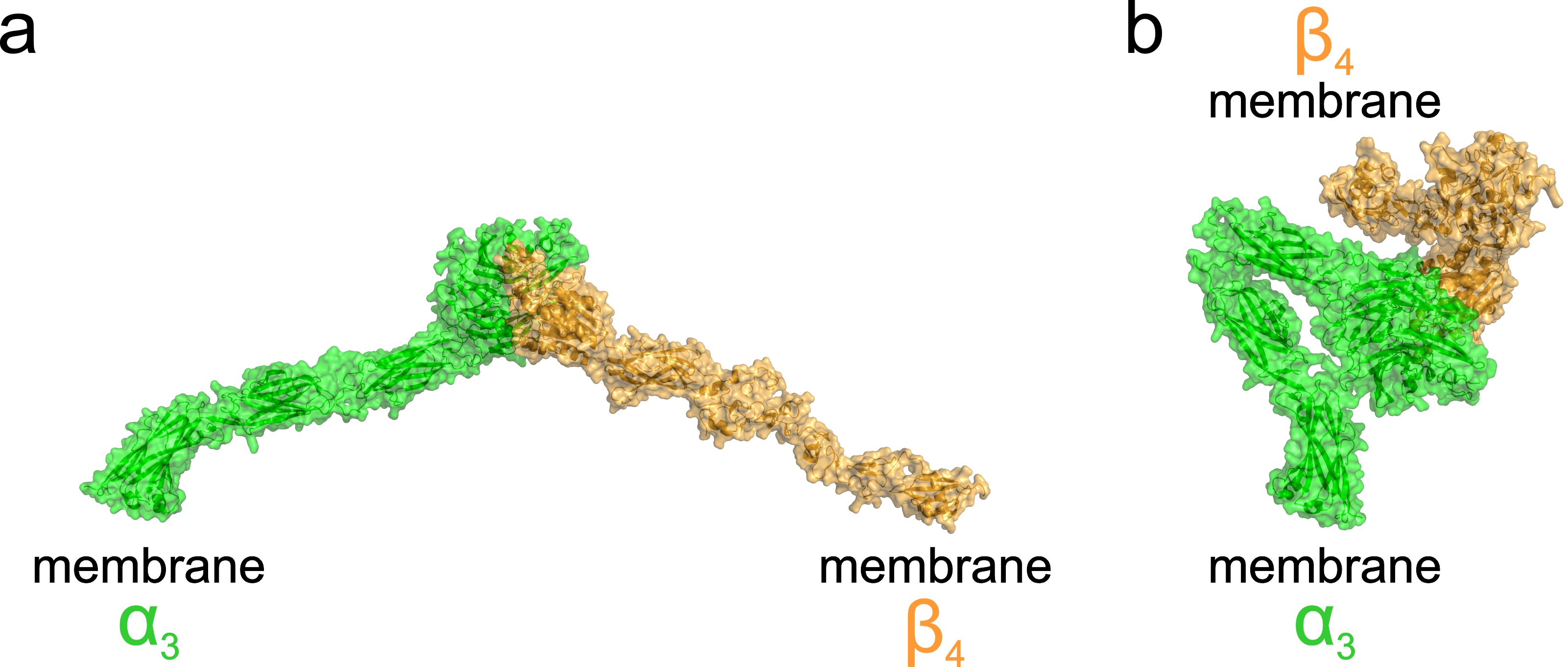

Supplement: Supplementary file 1 [file ijms-20-01004-s001.zip › Supplementary Fig 2.png]
